# InChI - the worldwide chemical structure standard

**DOI:** 10.1186/1758-2946-5-S1-P37

**Published:** 2013-03-22

**Authors:** Stephen Heller

**Affiliations:** 1 Gaithersburg, MD, USA

## 

Since its introduction in 2005 the IUAPC InChI chemical structure standard has become the international, worldwide standard for defined chemical structures. This presentation will describe the extensive use and dissemination of the InChI and InChIKey structure representations by and for the world-wide chemistry community, the chemical information community, and major publishers and disseminators of chemical and related scientific offerings in manuscripts and databases.

